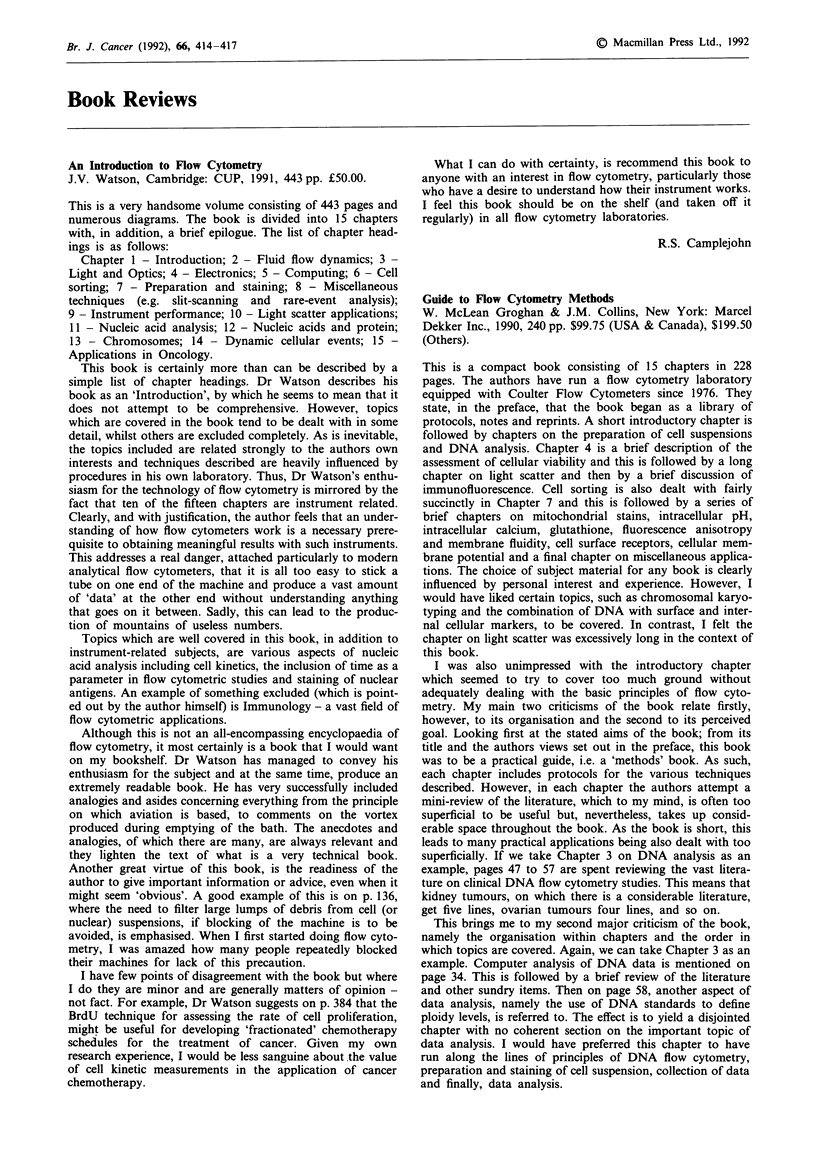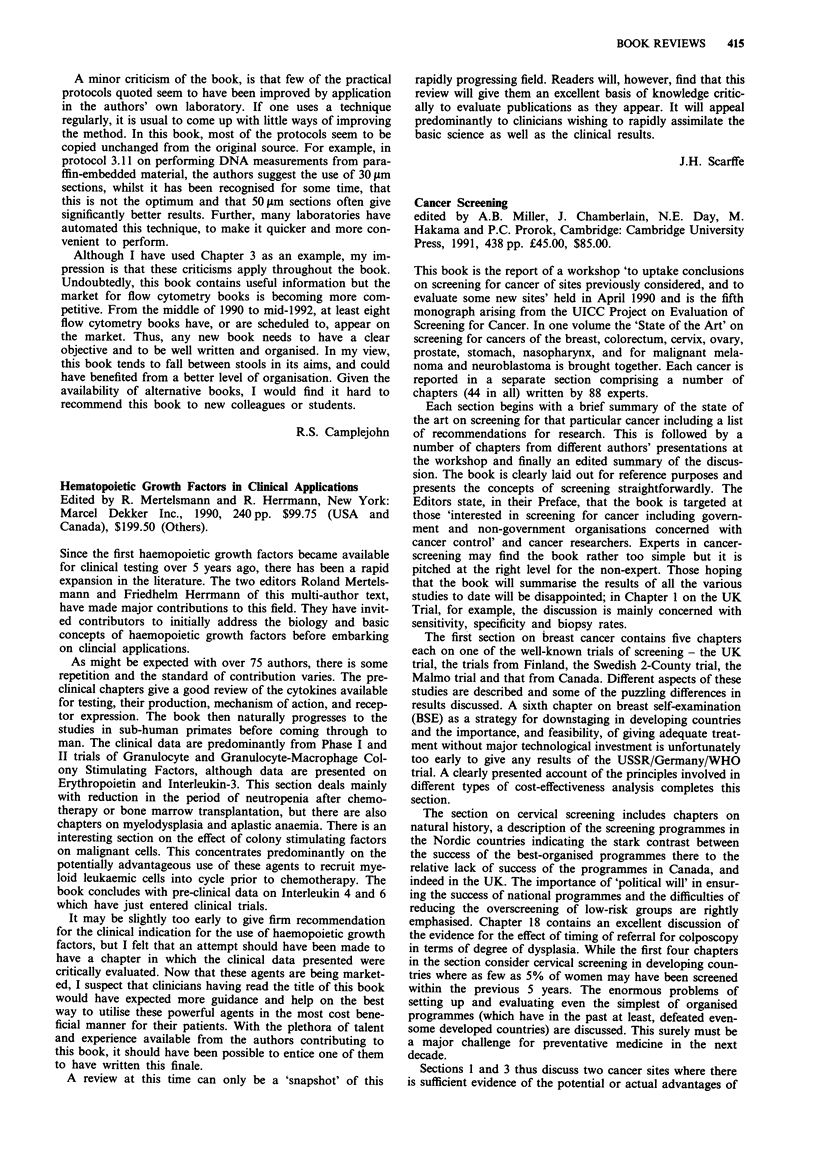# Guide to Flow Cytometry Methods

**Published:** 1992-08

**Authors:** R.S. Camplejohn


R.S. Camplejohn

**Guide to Flow Cytometry Methods**
W. McLean Groghan & J.M. Collins, New York: Marcel Dekker Inc., 1990, 240 pp. $99.75 (USA & Canada), $199.50 (Others).

This is a compact book consisting of 15 chapters in 228 pages. The authors have run a flow cytometry laboratory equipped with Coulter Flow Cytometers since 1976. They state, in the preface, that the book began as a library of protocols, notes and reprints. A short introductory chapter is followed by chapters on the preparation of cell suspensions and DNA analysis. Chapter 4 is a brief description of the assessment of cellular viability and this is followed by a long chapter on light scatter and then by a brief discussion of immunofluorescence. Cell sorting is also dealt with fairly succinctly in Chapter 7 and this is followed by a series of brief chapters on mitochondrial stains, intracellular pH, intracellular calcium, glutathione, fluorescence anisotropy and membrane fluidity, cell surface receptors, cellular membrane potential and a final chapter on miscellaneous applications. The choice of subject material for any book is clearly influenced by personal interest and experience. However, I would have liked certain topics, such as chromosomal karyotyping and the combination of DNA with surface and internal cellular markers, to be covered. In contrast, I felt the chapter on light scatter was excessively long in the context of this book.

I was also unimpressed with the introductory chapter which seemed to try to cover too much ground without adequately dealing with the basic principles of flow cytometry. My main two criticisms of the book relate firstly, however, to its organisation and the second to its perceived goal. Looking first at the stated aims of the book; from its title and the authors views set out in the preface, this book was to be a practical guide, i.e. a 'methods' book. As such, each chapter includes protocols for the various techniques described. However, in each chapter the authors attempt a mini-review of the literature, which to my mind, is often too superficial to be useful but, nevertheless, takes up considerable space throughout the book. As the book is short, this leads to many practical applications being also dealt with too superficially. If we take Chapter 3 on DNA analysis as an example, pages 47 to 57 are spent reviewing the vast literature on clinical DNA flow cytometry studies. This means that kidney tumours, on which there is a considerable literature, get five lines, ovarian tumours four lines, and so on.

This brings me to my second major criticism of the book, namely the organisation within chapters and the order in which topics are covered. Again, we can take Chapter 3 as an example. Computer analysis of DNA data is mentioned on page 34. This is followed by a brief review of the literature and other sundry items. Then on page 58, another aspect of data analysis, namely the use of DNA standards to define ploidy levels, is referred to. The effect is to yield a disjointed chapter with no coherent section on the important topic of data analysis. I would have preferred this chapter to have run along the lines of principles of DNA flow cytometry, preparation and staining of cell suspension, collection of data and finally, data analysis.

A minor criticism of the book, is that few of the practical protocols quoted seem to have been improved by application in the authors' own laboratory. If one uses a technique regularly, it is usual to come up with little ways of improving the method. In this book, most of the protocols seem to be copied unchanged from the original source. For example, in protocol 3.11 on performing DNA measurements from paraffin-embedded material, the authors suggest the use of 30 μm sections, whilst it has been recognised for some time, that this is not the optimum and that 50 μm sections often give significantly better results. Further, many laboratories have automated this technique, to make it quicker and more convenient to perform.

Although I have used Chapter 3 as an example, my impression is that these criticisms apply throughout the book. Undoubtedly, this book contains useful information but the market for flow cytometry books is becoming more competitive. From the middle of 1990 to mid-1992, at least eight flow cytometry books have, or are scheduled to, appear on the market. Thus, any new book needs to have a clear objective and to be well written and organised. In my view, this book tends to fall between stools in its aims, and could have benefited from a better level of organisation. Given the availability of alternative books, I would find it hard to recommend this book to new colleagues or students.